# Identification and Functional Analysis of Long Non-coding RNAs in Autism Spectrum Disorders

**DOI:** 10.3389/fgene.2020.00849

**Published:** 2020-09-16

**Authors:** Zhan Tong, Yuan Zhou, Juan Wang

**Affiliations:** ^1^Department of Biomedical Informatics, School of Basic Medical Sciences, Peking University, Beijing, China; ^2^Autism Research Center of Peking University Health Science Center, Peking University, Beijing, China

**Keywords:** long non-coding RNA, autism spectrum disorders, enrichment analysis, genome variants, drug prediction

## Abstract

Genetic and environmental factors, alone or in combination, contribute to the pathogenesis of autism spectrum disorder (ASD). Although many protein-coding genes have now been identified as disease risk genes for ASD, a detailed illustration of long non-coding RNAs (lncRNAs) associated with ASD remains elusive. In this study, we first identified ASD-related lncRNAs based on genomic variant data of individuals with ASD from a twin study. In total, 532 ASD-related lncRNAs were identified, and 86.7% of these ASD-related lncRNAs were further validated by an independent copy number variant (CNV) dataset. Then, the functions and associated biological pathways of ASD-related lncRNAs were explored by enrichment analysis of their three different types of functional neighbor genes (i.e., genomic neighbors, competing endogenous RNA (ceRNA) neighbors, and gene co-expression neighbors in the cortex). The results have shown that most of the functional neighbor genes of ASD-related lncRNAs were enriched in nervous system development, inflammatory responses, and transcriptional regulation. Moreover, we explored the differential functions of ASD-related lncRNAs in distinct brain regions by using gene co-expression network analysis based on tissue-specific gene expression profiles. As a set, ASD-related lncRNAs were mainly associated with nervous system development and dopaminergic synapse in the cortex, but associated with transcriptional regulation in the cerebellum. In addition, a functional network analysis was conducted for the highly reliable functional neighbor genes of ASD-related lncRNAs. We found that all the highly reliable functional neighbor genes were connected in a single functional network, which provided additional clues for the action mechanisms of ASD-related lncRNAs. Finally, we predicted several potential drugs based on the enrichment of drug-induced pathway sets in the ASD-altered biological pathway list. Among these drugs, several (e.g., amoxapine, piperine, and diflunisal) were partly supported by the previous reports. In conclusion, ASD-related lncRNAs participated in the pathogenesis of ASD through various known biological pathways, which may be differential in distinct brain regions. Detailed investigation into ASD-related lncRNAs can provide clues for developing potential ASD diagnosis biomarkers and therapy.

## Introduction

Autism spectrum disorders (ASDs) are a group of heterogeneous neurodevelopmental disorders characterized by deficits in reciprocal social interaction and communication, and restricted interests and repetitive stereotypical behavior, with male-to-female prevalence nearly 3:1 (Constantino and Charman, 2016; Loomes et al., 2017). Approximately 10% of individuals with ASD have an identifiable genetic cause according to increasing clinical genetics services (Yuen et al., 2017). But because of highly genetic and phenotypic heterogeneity, the exact mechanism of ASD pathophysiology remains elusive (Betancur, 2011; Parikshak et al., 2016; Iakoucheva et al., 2019). Long non-coding RNAs (lncRNAs), which are defined as non-coding transcripts with more than 200 nucleotides in length, perform diverse regulation functions through a variety of mechanisms, including cell cycle regulation, RNA processing and editing, molecular scaffold, chromatin remodeling, genome imprinting, miRNA sponges, and transcriptional regulation (Mercer and Mattick, 2013; Marchese et al., 2017). It has been shown that the mutation and dysregulation of lncRNAs are involved in a wide variety of diseases, including cancer, neurological disorders, and cardiovascular diseases (Uchida and Dimmeler, 2015; Bhan et al., 2017; Chen et al., 2017; Tang et al., 2017). lncRNAs are also emerging as an important component in normal brain development (Li et al., 2018). Therefore, a detail illustration of lncRNA and its function mechanism involved in ASD will greatly expand our understanding of the pathogenesis of ASD.

Twins and family studies have illustrated the predominant roles of genetic factors in the pathogenesis of ASD (Pinto et al., 2010; Parikshak et al., 2016; Iakoucheva et al., 2019). Although many studies addressing the genetic architecture of ASD have mainly focused on illustrating the roles of protein-coding genes, an increasing number of researchers are exploring the association between lncRNAs and the pathogenesis of ASD (Parikshak et al., 2016; Tang et al., 2017; Cogill et al., 2018). Most of the evidence, which characterized lncRNA dysregulation as an integral component of the transcriptomic signature of ASD, was derived from gene expression profiles of individuals with ASD (Ziats and Rennert, 2013; Wang et al., 2015; Gudenas et al., 2017). Only several lncRNAs associated with ASD have been identified by genome-derived evidence, and further explored in action mechanisms by loss-of-function or gain-of-function experiments, such as SHANK2-AS, MSNP1-AS, and BDNF-AS (DeWitt et al., 2016; Luo et al., 2018). In consideration of spatiotemporal-specific expression patterns, lncRNAs execute different functions and have unique gene expression patterns in distinct cellular conditions, therefore differentially expressed lncRNAs in a specific tissue could not reflect the global effects of dysregulated lncRNAs (Pramparo et al., 2015). Identification and functional analysis of genome-derived lncRNAs may complement these limitations.

Gene co-expression analysis and genomic neighbor region analysis are recognized as two traditional annotation ways of the functions and associated biological pathways of uncharacterized lncRNAs, which have a wide range of applications (Li et al., 2014a; Saha et al., 2017). Competing endogenous RNA (ceRNA) crosstalk, which refers to a hypothesis that all RNA transcripts can communicate with and regulate each other through competing to bind shared miRNAs, has been increasingly recognized as an important way to study gene functions and associated biological mechanisms (Tay et al., 2014). In this study, we first identified ASD-related lncRNAs based on the genomic variants detected in individuals with ASD from the previous twin study, which defined the discordant variations in monozygotic twin (DVMT) occurred in at least two twin pairs as putative ASD risk sites (Huang et al., 2019). These ASD-related lncRNAs were found to have less gene essentiality scores and greater gene expression specificities, and tended to have greater median expression in the brain tissues when compared to all profiled tissues. Then we explored the functions and associated mechanisms of ASD-related lncRNAs using enrichment analysis of their three distinct types of functional neighbor genes, including genomic neighbors, ceRNA neighbors, and gene co-expression neighbors (derived from gene expression profiles in the cortex). We also explored the distinct functions of ASD-related lncRNAs in different brain regions. Furthermore, a functional interaction network was constructed for the highly reliable functional neighbor genes of ASD-related lncRNAs. Finally, several drugs which have potential to intervene in the aberrant state of ASD in the biological pathway level were predicted.

## Materials and Methods

### Identifying Long Non-coding RNAs (lncRNAs) in Autism Spectrum Disorder

We obtained the genomic variant data of individuals with autism spectrum disorder (ASD) from the Huang et al. (2019) study, which identified the genomic variants in monozygotic twins discordant for ASD using genome-wide sequencing. Then the genomic coordinate data of reference genes from the UCSC genome browser^1^ (Casper et al., 2018) (i.e., known gene track) and the Ensembl database^2^ (Zerbino et al., 2018) were used to intersect the genomic variant data to identify long non-coding RNAs (lncRNAs) with overlapping mutations, which were so-called ASD-related lncRNAs in the following. These five gene types in the Ensembl database (i.e., antisense, bidirectional promoter lncRNA, lincRNA, non-coding, sense intronic) were taken as lncRNA subclasses in this study. Since the genomic annotation data we used before were lncRNA genes including exons and introns, we also used lncRNA exons only, or lncRNA Ensembl genes with 5kb upstream regions as promoters to overlap with disease-related mutations to screen potential ASD-related lncRNAs.

To explore the reliability of ASD-related lncRNAs obtained from the twins’ study, we downloaded the copy number variation (CNV) dataset from the Simons Foundation Autism Research Initiative (SFARI) database^3^ (Abrahams et al., 2013). All genomic coordinates of the CNV dataset were transferred to the latest genome assembly (hg38) using the UCSC liftOver tool, and the regions that failed to convert were discarded. Then we established the CNV-related lncRNAs by intersecting the transferred genomic coordinates of the CNV dataset and the genomic location data of reference genes.

We also searched the expression profiling data by high-throughput sequencing in the GEO database by using “autism,” “autism spectrum disorder” or “ASD” as keywords. GSE64018, which contains samples from postmortem brain tissues of both ASD individuals and healthy controls (12 vs 12), was chosen to calculate the differentially expressed genes (DEGs) in ASD. In this procedure, we chose fold-change >1.5 or <1/1.5 and *p*-value by the Wilcoxon rank sum test <0.05 as the criteria to identify DEGs. Only DEGs which met our definition of lncRNAs were retained.

### Gene Importance and Expression Specificity Analysis of ASD-Related lncRNAs

We downloaded gene importance score data from the Gene Importance Calculator (GIC) database,^4^ which defined gene essentiality scores of protein-coding genes and lncRNAs based on sequence features (Zeng et al., 2018). Zeng et al. constructed the GIC model based on the sequence features and logistic regression model, and defined the GIC score as the conditional probability *p* that a gene is essential (*Y* = 1) calculated by the respective logistical regression model. Then the Wilcoxon rank sum test was performed to investigate the GIC score characteristics of ASD-related lncRNAs.

The tissue-specific RNA-seq dataset was downloaded from the Genotype-Tissue Expression (GTEx) project,^5^ in which gene-level median TPM values are reported for 53 different tissue types (Ardlie et al., 2015). A brain tissue expression index was calculated for each gene by the median of the expression values in brain tissues divided by the median expression for all tissue types. On the basis of the RNA-seq dataset, the tissue specificity index τ was calculated for each gene based on the program proposed by Yanai et al. (2005). The Wilcoxon rank sum test was then performed to explore the brain tissue expression index and tissue specificity index characteristics of ASD-related lncRNAs.

### Weighted Gene Co-expression Network Analysis (WGCNA)

We constructed specific gene co-expression networks for different brain regions using the WGCNA package in R (v1.66) (Zhang and Horvath, 2005). To filter many genes with subtle expression or limited expression variation across different samples, only genes with top 75% median absolute deviation (MAD) across samples were retained. All genes and samples were then checked for excessive missing values, and the obvious outliers were excluded by sample hierarchical clustering. The function pickSoftThreshold provided by the WGCNA package was used to choose the proper soft thresholding power to approximate network scale-free topology. Afterward, an unsigned weighted network was created using the selected soft thresholding power and the Pearson correlation. Based on the gene expression profiles and associated sample annotation file from the GTEx database (see text footnote 5), a total of seven brain region specific gene co-expression networks (i.e., cortex, frontal cortex (BA9), anterior cingulate cortex (BA24), cerebellum, hippocampus, hypothalamus, and amygdala) were constructed, respectively.

### Establishment of Functional Neighbor Genes of ASD-Related lncRNAs

According to previous studies, there occur three complementary ways to predict the unknown function of lncRNA, including enrichment analysis of lncRNA’s genomic neighbors, competing endogenous RNAs (ceRNAs) neighbors, and gene co-expression neighbors. In this study, we chose the upstream and downstream 50 kb flanking region of the lncRNA gene as its neighbor region (Li et al., 2014a). Then BEDTools (Quinlan and Hall, 2010) was used to find the overlaps between the genomic coordinates of reference genes from the Ensembl database and the lncRNAs’ neighbor regions, and finally identified the genomic neighbors of ASD-related lncRNAs. Competing endogenous RNAs (ceRNAs), which share at least two regulating miRNAs with ASD-related lncRNAs, were retrieved from the starBase v2.0 database^6^ with the threshold of *p*-value and false discovery rate (FDR) corrected *p*-value ≤0.01, and with the pancancerNum parameter setting as 0 to avoid unwanted bias on the selected ceRNAs when studying disease other than cancers (Li et al., 2014b). These connected protein-coding genes were considered as the ceRNA neighbors of ASD-related lncRNAs. We speculated that the human cortex was more relevant to the pathophysiology of ASD compared to the other brain regions, based on much more ASD researches using the cortex samples (Willsey et al., 2013; Parikshak et al., 2016; Gudenas et al., 2017). So we obtained gene co-expression neighbors of ASD-related lncRNAs from the weighted gene co-expression network based on gene expression profiling of the cortex samples. For each functional neighbor gene set, only protein-coding genes were retained for the following analyses. The known ASD-associated genes from the AutDB and SFARI database were used to annotate these functional neighbor genes of ASD-related lncRNAs (Abrahams et al., 2013; Pereanu et al., 2018).

### Functional Analysis

For each functional neighbor gene set, we conducted gene ontology (GO) and KEGG pathway enrichment analysis using the DAVID web server^7^ (Huang Da et al., 2009a, b). For functional neighbor genes which occur in at least two among three functional neighbor categories (genomic neighbors, ceRNA neighbors, gene co-expression neighbors in the cortex), we explored the functional network of these genes using GeneMANIA^8^ (Franz et al., 2018). Notably, a more stringent threshold for filtering gene co-expression neighbors was used to obtain a reasonable number of highly reliable functional neighbor genes in the analysis of functional network.

### Potential Drug Prediction

We downloaded the drug-induced KEGG pathway dataset from the Drug-Path database^9^ (Zeng et al., 2015), which predicted drug-induced pathways based on the transcriptome downloaded from the CMap database. Then we grouped the KEGG pathways by the same drugs to generate the specific drug-induced KEGG pathway sets. The Fisher’s exact test was used to find the overrepresentation of each drug-induced KEGG pathway set (i.e., potential drug) in the altered KEGG pathway list obtained from functional analysis of ASD-related lncRNAs. To obtain the altered KEGG pathway list with an appropriate number, we used FDR ≤0.5 as the filtering threshold. All human KEGG pathways included in the KEGG database^10^ (Kanehisa et al., 2017) were taken as the background.

## Results

### Identification and Characterization of Long Non-coding RNAs in Autism Spectrum Disorders

**FIGURE 1:**
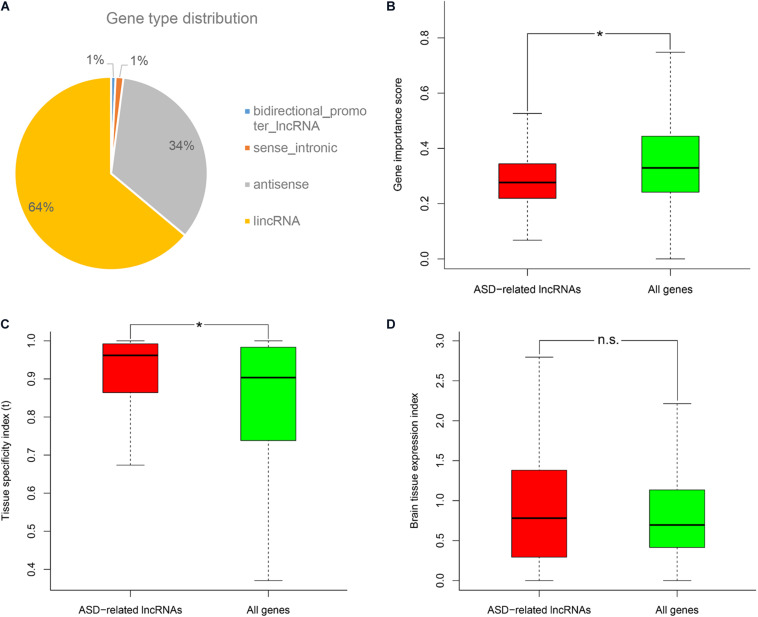
Gene type distribution and characteristics analysis of ASD-related lncRNAs. **(A)** Pie chart displays the distribution of ASD-related lncRNAs in different gene types. Comparison of ASD-related lncRNAs with the background genes in the **(B)** gene importance score, **(C)** tissue specificity index τ, and **(D)** brain tissue expression index. *P-value <10E-5 from Wilcoxon test. N.s. represents no statistical significance.

Firstly, we downloaded the genomic variant dataset of individuals with autism spectrum disorders (ASDs) from the previous study, which included single nucleotide variants (SNVs), small insertions or deletions (Indels), and copy number variants (CNVs) (Huang et al., 2019). Then the genomic locations of reference genes were scanned to identify ASD-related long non-coding RNAs (lncRNAs) which had overlapped mutations associated with ASD. In total, 532 ASD-related lncRNAs were identified and further classified into several categories based on gene type annotations from the Ensembl database (Supplementary Table 1; Zerbino et al., 2018). We found that more than a half of ASD-related lncRNAs came from genome intergenic regions, and lncRNAs which were generated from the antisense strand of protein-coding genes, also occupied a large proportion (Figure 1A). By using different genomic annotations of lncRNAs (i.e., lncRNA genes, lncRNA exons, lncRNA genes with promoters), we analyzed the overlapping relationships of the respectively identified ASD-related lncRNA lists (Supplementary Figure 1). We found that ASD-related lncRNAs identified using lncRNA genes as reference genomic annotations included 100 and 83.52% of the ASD-related lncRNAs identified using lncRNA exons and lncRNA genes with promoters, respectively. So we used the ASD-related lncRNAs identified using lncRNA genes as reference genomic annotations for the following analysis. Since the original genomic variant dataset was derived from small samples and single cohort, we also validated these ASD-related lncRNAs with the CNV dataset from the SFARI database (Abrahams et al., 2013). A total of 86.7% of ASD-related lncRNAs were shown to overlap with at least one ASD-associated CNV. To avoid the overestimation caused by possible consistence occurring in the CNV datasets, we excluded the CNV data from our genomic variant dataset and re-identified ASD-related lncRNAs using the previous procedure. We found that 80.42% of the newly identified ASD-related lncRNAs were still supported by at least one ASD-associated CNV. Through the characteristics analyses, ASD-related lncRNAs were shown to have lower gene importance, and higher tissue expression specificity compared to the background genes (Wilcoxon rank sum test, *P*-value = 1.61E-18 and 2.79E-13, respectively) (Figures 1B,C). Moreover, ASD-related lncRNAs seem to have a bigger brain tissue expression index (i.e., the ratio of median gene expression in brain tissues vs all profiled tissues) compared to the background, but no significant difference was detected (Figure 1D).

### Functional Enrichment Analysis of ASD-Related lncRNAs

**FIGURE 2:**
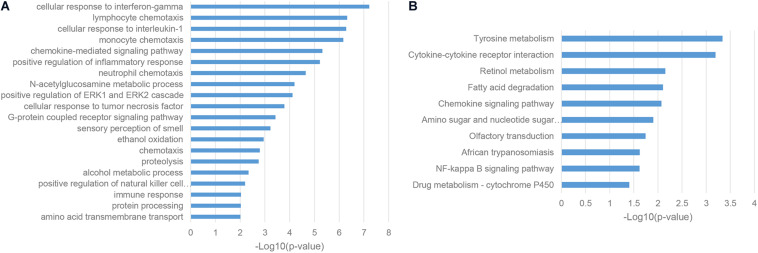
Enriched functional terms of genomic neighbors of ASD-related lncRNAs. **(A)** The top 20 gene ontology biological process (GO BP) terms with *p*-value <0.05. **(B)** KEGG pathway terms with *p*-value <0.05.

To characterize the functions and associated biological pathways of ASD-related lncRNAs, it is an important step to find their functional-relevant protein-coding genes. LncRNAs are recognized as potential cis-regulators of their genomic neighbor genes (Bao et al., 2019), so it is a reasonable way to predict the functions and implicated biological pathways of ASD-related lncRNAs based on their genomic neighbors. Gene ontology (GO) enrichment analysis of genomic neighbors has shown that most of these genes were enriched in a series of immune pathways, including cellular response to interferon-gamma, cellular response to interleukin-1, and positive regulation of the inflammatory response (Figure 2A; Estes and McAllister, 2015). KEGG pathway analysis illustrated the enrichment in the tyrosine metabolism pathway, which was consistent with the fact that MET receptor tyrosine kinase (RTK) is an autism risk factor (Figure 2B; Ma et al., 2019). Together, these results indicated that ASD-related lncRNAs may be implicated in the pathogenesis of autism partly through immune response processes.

**FIGURE 3:**
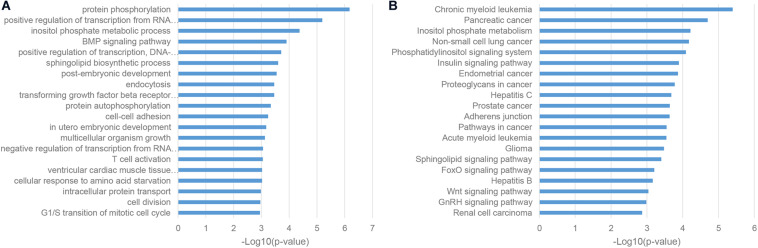
Enriched functional terms of competing endogenous RNA (ceRNA) neighbors of ASD-related lncRNAs. **(A)** The top 20 GO BP terms with *p*-value <0.05. **(B)** Top 20 KEGG pathway terms with *p*-value <0.05.

Given the fact that lncRNAs can regulate protein-coding mRNAs through the mechanism of miRNA sponges (Tay et al., 2014), we conducted the enrichment analysis of competing endogenous RNA (ceRNA) neighbors of ASD-related lncRNAs. We found that most of these genes were enriched in transcriptional regulation, such as positive regulation of transcription from RNA polymerase II promoter and positive regulation of transcription, DNA-templated (Figure 3A; De Rubeis et al., 2014). Furthermore, KEGG pathway analysis uncovered the implication of the insulin signaling pathway, Glioma and Wnt signaling pathway, which was consistent with previous reports (Figure 3B; Parikshak et al., 2016; Iakoucheva et al., 2019).

**FIGURE 4:**
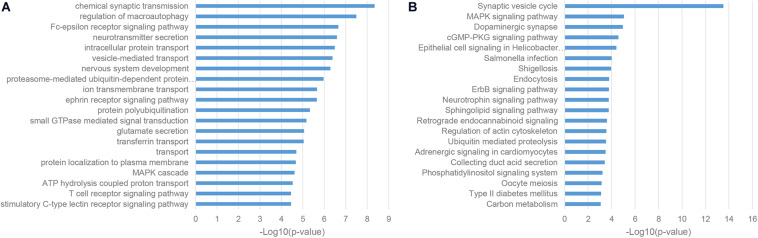
Enriched functional terms of gene co-expression neighbors of ASD-related lncRNAs. **(A)** The top 20 GO BP terms with *p*-value <0.05. **(B)** Top 20 KEGG pathway terms with *p*-value <0.05.

As a classic way for predicting the function of uncharacterized genes, we also performed the enrichment analysis of the gene co-expression neighbors of ASD-related lncRNAs in the human cortex, which was widely accepted as the most relevant tissue in the pathogenesis of ASD. As shown in Figures 4A,B, gene co-expression neighbors have shown the most relevance with the neuropathological mechanisms of ASD among these three categories of functional neighbor genes. Most of gene co-expression neighbors were enriched in the nervous system associated biological processes, such as chemical synaptic transmission, neurotransmitter secretion, and nervous system development from GO biological process (BP) terms, synaptic vesicle cycle and dopaminergic synapse from KEGG pathways (De Rubeis et al., 2014; Pramparo et al., 2015; Huang et al., 2019). Notably, the MAPK signaling pathway was identified by both enrichment analysis categories, which were inferred to be involved in the pathogenesis of ASD through the regulation of cell-proliferation pathways in brain development (Iakoucheva et al., 2019).

All these results provided evidence of the involvement of ASD-related lncRNAs in the pathogenesis of autism. It also implied that ASD-related lncRNAs may participate in various ASD implicated biological pathways through multiple regulation mechanisms when considering the complementary enrichment analysis results of different types of functional neighbor genes. These results also prompted some additional biological pathways potentially associated with ASD. For example, proteolysis and/or protein ubiquitination were uncovered in the enrichment analysis of both genomic and gene co-expression neighbors, but the detailed mechanism of its involvement in autism remains unclear. Moreover, we tried to explore whether our identified ASD-related lncRNAs were more relevant to the pathogenesis of ASD compared to the background lncRNAs. Generally, we found that the functional neighbors of ASD-related lncRNAs tended to have a higher proportion of known ASD-associated genes when compared to those of the background lncRNAs (Wilcoxon rank sum test, *P*-value = 8.14E-3).

In addition, we noticed that it was interesting to analysis the novelty and significance of our genomic variant-derived ASD-related lncRNAs. So we obtained 465 differentially expressed lncRNAs in the brain tissues in ASD using the GSE64018 dataset. We found there were 18 ASD-related lncRNAs overlapping with the differentially expressed lncRNA list (Supplementary Figure 2). Since the numbers of genomic and ceRNA neighbors of these 18 lncRNAs were small, we only performed the functional enrichment analysis for their gene co-expression neighbors. For the comparison, the similar functional enrichment analysis was also conducted for the non-overlapping ASD-related lncRNAs. The GO BP enrichment analysis results showed that the gene co-expression neighbors of 18 overlapping lncRNAs were mainly enriched in transport-related pathways, including vesicle-mediated transport, neurotransmitter secretion, and chemical synaptic transmission, while the remaining 514 lncRNAs’ were also shown to be enriched in nervous system development besides these biological processes (Supplementary Figures 3, 4). KEGG pathway analysis has showed similar results (Supplementary Figures 3, 4). These results suggested that genomic variants-derived ASD-related lncRNAs had comparable novelty and potential to illustrate the pathogenesis mechanism of ASD.

### Differentially Enriched Functional Terms of ASD-Related lncRNAs in Different Brain Regions

**FIGURE 5:**
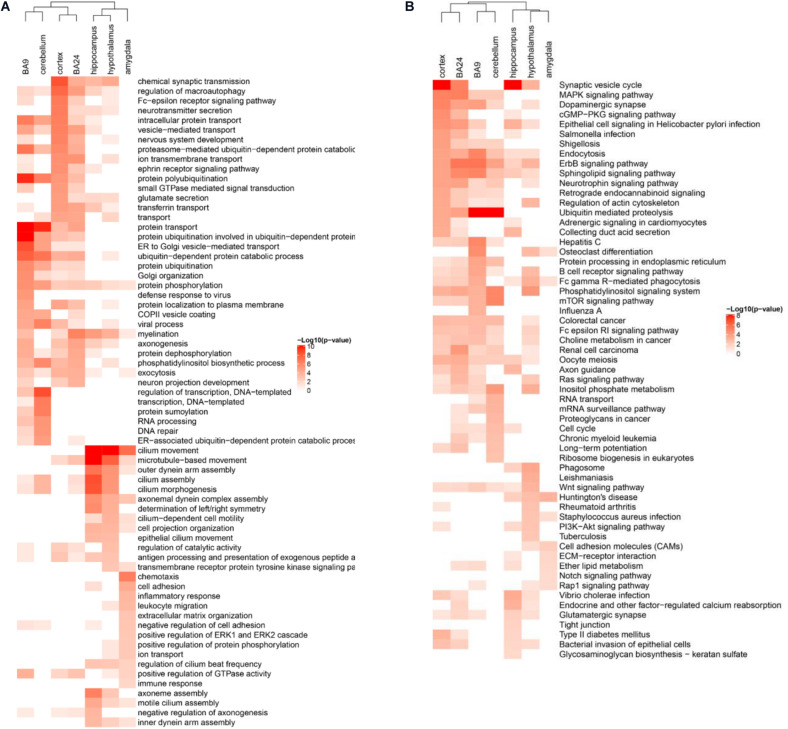
Differentially enriched functional terms of ASD-related lncRNAs in distinct brain regions. Heatmaps display enriched **(A)** GO BP terms and **(B)** KEGG pathways of gene co-expression neighbors of ASD-related lncRNAs in different brain regions. Only the top 15 terms with *p*-value <0.05 in each brain region were depicted for both functional categories.

During the gene co-expression analysis in the human cortex, we noticed that it was interesting to illustrate whether ASD-related lncRNAs executed similar functions in different brain regions. Several brain regions have been reported to be associated with the pathogenesis of ASD, such as cortex, frontal cortex (BA9), anterior cingulate cortex (BA24), cerebellum, hippocampus, hypothalamus, and amygdala (Amaral et al., 2008; Parikshak et al., 2016; Ma et al., 2019; Zhang et al., 2019). So we constructed specific weighted gene co-expression networks for these distinct brain regions, and then performed enrichment analysis using the pipeline described before, respectively. Actually, the results of enrichment analysis have shown the differential functions of ASD-related lncRNAs as a class in distinct brain regions (Figures 5A,B). Most of the gene co-expression neighbors of ASD-related lncRNAs in the cerebellum were enriched in transcriptional regulation and DNA repair, while neighbors in the cortex were enriched in a variety of nervous system related functions, consistent with the important roles of the cortex in the pathogenesis of ASD. Interestingly, neighbors in BA9 and BA24 have shown significant differences in enriched function terms. Most of the gene co-expression neighbors in BA9 were enriched in protein ubiquitination and ubiquitin-mediated proteolysis, while gene co-expression neighbors in BA24 have also shown enrichment in several neuron related functions, including myelination and axonogenesis. Previous studies have illustrated an excess of 67% neurons in the prefrontal, and a quantitative gradient of brain overgrowth from anterior/frontal to posterior in the majority of individuals with ASD (Pramparo et al., 2015). One plausible mechanism would be the degree of dysregulation of ubiquitin-proteasome dependent degradation resulting in the gradient of neural cell growth (Crider et al., 2014). Furthermore, most of the gene co-expression neighbors in the hippocampus and hypothalamus were also enriched in several nervous system functions, while neighbors in the amygdala were enriched in cell adhesion and inflammatory response (Estes and McAllister, 2015; Pramparo et al., 2015; Parikshak et al., 2016; Iakoucheva et al., 2019).

### Network Analysis of Highly Reliable Functional Neighbor Genes of ASD-Related lncRNAs

**FIGURE 6:**
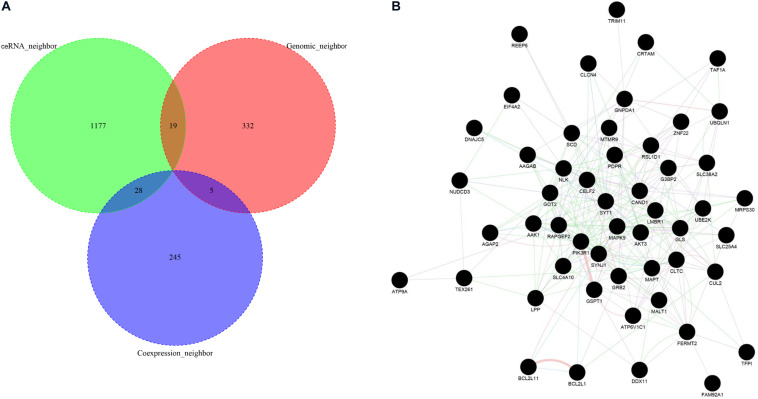
Functional network analysis of high-reliable functional neighbor genes of ASD-related lncRNAs. **(A)** The Venn plot displays the intersection of three types of functional neighbor genes, i.e., genomic neighbors, competing endogenous RNA (ceRNA) neighbors, and gene co-expression neighbors. **(B)** The network graph depicts the functional interaction relationships between each high-reliable functional neighbor gene pair.

To obtain the highly reliable functional neighbor genes of ASD-related lncRNAs, we intersected the three types of functional neighbor genes (i.e., genomic neighbors, ceRNA neighbors, and gene co-expression neighbors filtered with a more stringent threshold in the cortex), and only retained the functional neighbor genes that occurred in at least two of these three categories (Figure 6A). Using the GeneMANIA webserver, we found that all these highly reliable functional neighbor genes were connected to each other in a single functional network, which included 59.6% co-expression, 21.9% physical interaction, 9.0% co-localization, 5.0% pathway, and 4.4% genetic interaction links (Figure 6B). In this functional network, several genes were known to be associated with ASD, including SYT1, DDX11, AGAP2, SLC4A10, and SYNJ1 (Abrahams et al., 2013). Moreover, these genes were related to several biological pathways, such as neurotransmitter secretion and transport, regulation of neurotransmitter levels, and MAP kinase activity.

### Potential Drugs for Intervening in Aberrant Biological Pathways in ASD

Considering that heterogeneous genetic mutations of ASD were shown to converge in the common biological pathways (Betancur, 2011; Parikshak et al., 2016), we wondered whether there were some drugs which had the potential to intervene in the aberrant state of ASD at the biological pathway level. So we used Fisher’s exact test to measure the overrepresented drug-induced KEGG pathway set (i.e., potential drug) in the altered KEGG pathway list obtained from the functional analysis of ASD-related lncRNAs. Several enriched drugs are predicted to be potentially associated with ASD (Table 1). For example, amoxapine clinically used for depression treatment, has been demonstrated to have treatment benefits for interfering behaviors in individuals with ASD (false discovery rate (FDR) adjusted *p*-value = 0.0236) (Craven-Thuss and Nicolson, 2003). Piperine was experimentally supported to have the ability to reduce oxidative stress, elevate brain glutathione, and improve behavior in a VPA (valproic acid) -induced ASD mice model (Ornoy et al., 2019). Diflunisal was shown to reduce the progression of neurological impairment and maintain quality of life in a randomized clinical trial for familial amyloid polyneuropathy (Berk et al., 2013). Eticlopride, which was firstly developed for the treatment of schizophrenia, now is used in the study of the roles of D2-like receptors in schizophrenia and other brain diseases (Martelle and Nader, 2008). To explore the drug action targets of these potential drugs, we conducted enrichment analysis with the DrugPattern tool by using these ten enriched drugs as input (Huang et al., 2018). We found that the associated drug targets mainly included dopamine receptors, sodium-dependent serotonin transporters, adrenergic receptors, and 5-hydroxytryptamine receptors (Supplementary Table 2).

**TABLE 1:** The top 10 enriched drugs for potentially intervening in aberrant KEGG pathways in ASD.

Drug name	*P*-value	FDR	Description
Amoxapine	8.91e-05	0.0236	Antidepressant, Serotonin-noradrenaline reuptake inhibitor
Diflunisal	9.62e-05	0.0236	Analgesic, Anti-inflammatory, COX inhibitor
Budesonide	3.29e-04	0.0605	Antiasthmatic, Anti-inflammatory, Glucocorticoid receptor agonist
Iopromide	4.97e-04	0.0649	Diagnostic aid (radiopaque medium)
Ethoxyquin	6.66e-04	0.0649	UDP-glucuronosyltransferase activator, neuroprotective agent, Hsp90 inhibitor, genotoxin, food antioxidant.
Cyclizine	6.86e-04	0.0649	Anti-emetic, H1 receptor antagonist
PNU-0293363	7.04e-04	0.0649	Not found
Eticlopride	1.05e-03	0.0843	Dopamine Antagonists, Neurotransmitter Agents
Piperine	1.14e-03	0.0843	NF-kappaB inhibitor
Nomegestrol	1.32e-03	0.0882	Ovulation inducing agent, Progesterone receptor agonist

*P-values were calculated with Fisher’s exact test. FDR represents the p-values adjusted by false discovery rate correction. The description information was retrieved from the KEGG drug (Kanehisa et al., 2017), DrugBank (Wishart et al., 2018), and PubChem (Kim et al., 2019) databases.*

## Discussion

LncRNAs, which constitute a large proportion of transcriptome, are increasingly recognized as the integral component of many fundamental biological processes and various disease pathogeneses. However, to our knowledge, limited efforts have been made to systematically characterize the functions and associated biological pathways of genome-level ASD-related lncRNAs. In this study, we first identified ASD-related lncRNAs using the genomic variant data in individuals with ASD downloaded from the previous study. Then the enrichment analysis of three types of functional neighbor genes provided abundant evidence of the involvement of ASD-related lncRNAs in the pathogenesis of autism through various biological pathways, including nervous system development, chemical synaptic transmission, transcriptional regulation, and immune pathway. The regulation of neurodevelopment was considered the center of ASD pathogenesis. De Rubeis et al. found that many of the genes damaged by risk variation in ASD encoded proteins for synaptic, transcriptional, and chromatin remodeling pathways, which include the voltage-gated ion channels regulating propagation of action potentials, pace-making, and excitability-transcription coupling, as well as histone-modifying enzymes and chromatin remodelers (De Rubeis et al., 2014). Increasing evidence has shown immune dysregulation in individuals with ASD, and several molecular signaling pathways have been identified to link immune activation to ASD phenotypes, including pathways downstream of cytokines, hepatocyte growth factor receptors (MET), MHCI molecules, microglia, and complement factors (Estes and McAllister, 2015). Moreover, several known ASD risk genes encode immune-related risk factors. Functional network analysis of the highly reliable functional neighbor genes of ASD-related lncRNAs (see more in Methods), which depicted a connected component, further implied that these ASD-related lncRNAs participated in common biological pathways through possible gene expression regulation mechanisms, such as neurotransmitter secretion and transport, regulation of neurotransmitter levels, and MAP kinase activity. Specific abnormalities of the glutamate neurotransmitter system have been detected in individuals with ASD through gene expression profiling analysis in mRNA and the protein level (Purcell et al., 2001).

Since many studies predicted potential drugs which could reverse expression profiles of disease, it is reasonable to consider this in the pathway level. Based on these altered KEGG pathways obtained from the functional analysis of ASD-related lncRNAs, we also predicted ten drugs with the potential to intervene in the disease status in the biological pathway level. As described before, several drugs has been shown to have treatment benefits for interfering behavior in individuals with ASD, or have ability to reduce oxidative stress, such as amoxapine and piperine (Craven-Thuss and Nicolson, 2003; Ornoy et al., 2019). Notably, immune dysregulation or inflammation, oxidative stress, mitochondrial dysfunction, and environmental toxicant exposures were regarded as four major areas of physiological and metabolic abnormalities in ASD and other psychiatric disorders (Rossignol and Frye, 2012). It was also worth mentioning that some other predicted drugs were experimentally validated to have efficacy in the treatment of other psychiatric disorders, such as diflunisal and eticlopride (Martelle and Nader, 2008; Berk et al., 2013). The enrichment analysis result of these ten predicted drugs uncovered several enriched drug targets including dopamine receptors, sodium-dependent serotonin transporters, adrenergic receptors, and 5-hydroxytryptamine receptors, which may provide useful information for drug development. For example, extended-release guanfacine, an alpha 2A/B adrenergic receptor agonist, was shown to have ability to reduce hyperactivity, impulsiveness, and distractibility in children with ASD (Scahill et al., 2015). However, the efficacy and exact mechanism of these predicted drugs for the treatment of ASD still need to be experimentally explored.

In the meanwhile, multiple brain regions have been shown to be involved in the pathogenesis of autism, but knowledge of the exact molecular mechanism and its difference in distinct brain regions are still limited. Interestingly, gene co-expression analysis in each brain region revealed the differential functions and associated biological pathways of ASD-related lncRNAs in distinct brain regions. As illustrated by the enrichment analysis, gene co-expression neighbors in the cortex tended to be mostly enriched in nervous system related functions, while gene co-expression neighbors in the cerebellum were enriched in transcription regulation and DNA repair. It was consistent with previous reports that a significant differential gene expression and differential splicing signal over background was found in the cortex, but not in the cerebellum (Parikshak et al., 2016). Willsey et al. (2013) implicated human midfetal layer 5/6 cortical projection neurons in the pathogenesis of ASD using co-expression networks. Gene co-expression neighbors in the frontal cortex (BA9) have shown more enrichment in ubiquitin-mediated proteolysis, while those in anterior cingulate cortex (BA24) tended to be enriched in myelination and axonogenesis. Most of the gene co-expression neighbors in the amygdala were enriched in cell adhesion and inflammatory response. Previous studies of large CNVs in ASD implicated several pathways in ASD pathogenesis, including synaptic transmission, transcriptional regulation, chromatin remodeling, translational regulation, ion transport, and cell adhesion (Iakoucheva et al., 2019). Together, these results provide insight for studying the differential functions of ASD-related lncRNAs in distinct brain regions.

Though these results greatly expand our understanding of the roles of lncRNAs in the genetic architecture of ASD, there are also some limitations in this study. Firstly, the genomic variant data, which was downloaded from the Huang et al. (2019) study, only included three pairs of monozygotic twins discordant for ASD. Statistical associations of these genomic variants with ASD have not been explored properly and the cohort studied was single, which may cause possible errors in the detection of ASD-related lncRNAs or restrict the translation of the results to other cohorts. Moreover, we still lack evidence from transcriptome to validate the results we obtained based on the genomic alternations. Given the highly genetic heterogeneous of ASD, multi-layer omics datasets would bridge genomic variants to transcriptome consequences, and provide additional clues for ASD pathogenesis and therapy.

In summary, we identified ASD-related lncRNAs and comprehensively explored their functions and associated biological pathways. We found that ASD-related lncRNAs participated in the pathogenesis of ASD through various biological pathways, which may also be different in distinct brain regions. Through functional network analysis, the highly reliable functional neighbor genes were shown to be connected in a single component, which further implied that ASD-related lncRNAs participated in common biological pathways, such as neurotransmitter secretion and transport, regulation of neurotransmitter levels, and MAP kinase activity. Several drugs which had potential to intervene in the disease status of ASD in the biological pathway level, were predicted. In a word, taking lncRNAs into the framework of genetic architecture of ASD could draw a more comprehensive landscape of genetic factors and their interplays, and provide new approaches for ASD diagnosis and therapy.

## Data Availability Statement

All datasets presented in this study are included in the article/Supplementary Material.

## Author Contributions

JW conceived and designed this study and revised the manuscript. YZ provided valuable comments and suggestions. ZT performed the analysis and drafted the manuscript. All authors read and approved the final manuscript.

## Conflict of Interest

The authors declare that the research was conducted in the absence of any commercial or financial relationships that could be construed as a potential conflict of interest.

## Footnotes

1 https://genome.ucsc.edu/

2 http://www.ensembl.org/

3 https://gene.sfari.org/autdb/GS_Home.do, SFARI-Gene_cnvs_06-20-2019release

4 http://www.cuilab.cn/gic

5 http://gtexportal.org, 2016-01-15_v7_RNASeQCv1.1.8 release

6 http://starbase.sysu.edu.cn/

7 https://david.ncifcrf.gov/

8 http://genemania.org/

9 http://www.cuilab.cn/drugpath

10 https://www.genome.jp/kegg/

## References

[B1] Abrahams B. S. Arking D. E. Campbell D. B. Mefford H. C. Morrow E. M. Weiss L. A. (2013). SFARI Gene 2.0*: a community-driven knowledgebase for the autism spectrum disorders (ASDs)*. *Mol. Autism.* 4:36. 10.1186/2040-2392-4-36 24090431 PMC3851189

[B2] Amaral D. G. Schumann C. M. Nordahl C. W. (2008). Neuroanatomy of autism. *Trends Neurosci.* 31 137–145. 18258309 10.1016/j.tins.2007.12.005

[B3] Ardlie K. G. Deluca D. S. Segrè A. V. Sullivan T. J. Young T. R. (2015). Human genomics. *The Genotype-Tissue Expression (GTEx) pilot analysis: multitissue gene regulation in humans*. *Science* 348 648–660. 25954001 10.1126/science.1262110 PMC4547484

[B4] Bao Z. Yang Z. Huang Z. Zhou Y. Cui Q. Dong D. (2019). LncRNADisease 2.0*: an updated database of long non-coding RNA-associated diseases*. *Nucleic Acids Res.* 47 D1034–D1037. 30285109 10.1093/nar/gky905 PMC6324086

[B5] Berk J. L. Suhr O. B. Obici L. Sekijima Y. Zeldenrust S. R. Yamashita T. (2013). Repurposing diflunisal for familial amyloid polyneuropathy: a randomized clinical trial. *Jama* 310 2658–2667. 10.1001/jama.2013.283815 24368466 PMC4139164

[B6] Betancur C. (2011). Etiological heterogeneity in autism spectrum disorders: more than 100 genetic and genomic disorders and still counting. *Brain Res.* 1380 42–77. 10.1016/j.brainres.2010.11.078 21129364

[B7] Bhan A. Soleimani M. Mandal S. S. (2017). Long Noncoding RNA and Cancer: A New Paradigm. *Cancer Res.* 77 3965–3981. 10.1158/0008-5472.can-16-2634 28701486 PMC8330958

[B8] Casper J. Zweig A. S. Villarreal C. Tyner C. Speir M. L. Rosenbloom K. R. (2018). The UCSC Genome Browser database: 2018 update. *Nucleic Acids Res.* 46 D762–D769. 29106570 10.1093/nar/gkx1020 PMC5753355

[B9] Chen X. Yan C. C. Zhang X. You Z. H. (2017). Long non-coding RNAs and complex diseases: from experimental results to computational models. *Brief Bioinform.* 18 558–576. 27345524 10.1093/bib/bbw060 PMC5862301

[B10] Cogill S. B. Srivastava A. K. Yang M. Q. Wang L. (2018). Co-expression of long non-coding RNAs and autism risk genes in the developing human brain. *BMC Syst. Biol.* 12:91. 10.1186/s12918-018-0639-x 30547845 PMC6293492

[B11] Constantino J. N. Charman T. (2016). Diagnosis of autism spectrum disorder: reconciling the syndrome, its diverse origins, and variation in expression. *Lancet Neurol.* 15 279–291. 10.1016/s1474-4422(15)00151-9 26497771

[B12] Craven-Thuss B. Nicolson R. (2003). Amoxapine treatment of interfering behaviors in autistic disorder. *J. Am. Acad. Child. Adolesc. Psych.* 42 515–516. 10.1097/01.chi.0000046827.95464.48 12707553

[B13] Crider A. Pandya C. D. Peter D. Ahmed A. O. Pillai A. (2014). Ubiquitin-proteasome dependent degradation of GABAAalpha1 in autism spectrum disorder. *Mol. Autism.* 5:45. 10.1186/2040-2392-5-45 25392730 PMC4228821

[B14] De Rubeis S. He X. Goldberg A. P. Poultney C. S. Samocha K. Cicek A. E. (2014). Synaptic, transcriptional and chromatin genes disrupted in autism. *Nature* 515 209–215. 25363760 10.1038/nature13772 PMC4402723

[B15] DeWitt J. J. Grepo N. Wilkinson B. Evgrafov O. V. Knowles J. A. Campbell D. B. (2016). Impact of the Autism-Associated Long Noncoding RNA MSNP1AS on Neuronal Architecture and Gene Expression in Human Neural Progenitor Cells. *Genes* 7:76. 10.3390/genes7100076 27690106 PMC5083915

[B16] Estes M. L. McAllister A. K. (2015). Immune mediators in the brain and peripheral tissues in autism spectrum disorder. *Nat. Rev. Neurosci.* 16 469–486. 10.1038/nrn3978 26189694 PMC5650494

[B17] Franz M. Rodriguez H. Lopes C. Zuberi K. Montojo J. Bader G. D. (2018). GeneMANIA update 2018. *Nucleic Acids Res.* 46 W60–W64. 29912392 10.1093/nar/gky311 PMC6030815

[B18] Gudenas B. L. Srivastava A. K. Wang L. (2017). Integrative genomic analyses for identification and prioritization of long non-coding RNAs associated with autism. *PLoS One* 12:e0178532. 10.1371/journal.pone.0178532 28562671 PMC5451068

[B19] Huang C. Yang W. Wang J. Zhou Y. Geng B. Kararigas G. (2018). The DrugPattern tool for drug set enrichment analysis and its prediction for beneficial effects of oxLDL on type 2 diabetes. *J. Genet. Genom.* 45 389–397. 10.1016/j.jgg.2018.07.002 30054214

[B20] Huang Y. Zhao Y. Ren Y. Yi Y. Li X. Gao Z. (2019). Identifying Genomic Variations in Monozygotic Twins Discordant for Autism Spectrum Disorder Using Whole-Genome Sequencing. *Mol. Ther. Nucleic Acids* 14 204–211. 10.1016/j.omtn.2018.11.015 30623854 PMC6325071

[B21] Huang Da W. Sherman B. T. Lempicki R. A. (2009a). Bioinformatics enrichment tools: paths toward the comprehensive functional analysis of large gene lists. *Nucleic Acids Res.* 37 1–13. 10.1093/nar/gkn923 19033363 PMC2615629

[B22] Huang Da W. Sherman B. T. Lempicki R. A. (2009b). Systematic and integrative analysis of large gene lists using DAVID bioinformatics resources. *Nat. Protoc.* 4 44–57. 10.1038/nprot.2008.211 19131956

[B23] Iakoucheva L. M. Muotri A. R. Sebat J. (2019). Getting to the Cores of Autism. *Cell* 178 1287–1298. 10.1016/j.cell.2019.07.037 31491383 PMC7039308

[B24] Kanehisa M. Furumichi M. Tanabe M. Sato Y. Morishima K. (2017). KEGG: new perspectives on genomes, pathways, diseases and drugs. *Nucleic Acids Res.* 45 D353–D361. 27899662 10.1093/nar/gkw1092 PMC5210567

[B25] Kim S. Chen J. Cheng T. Gindulyte A. He J. He S. (2019). PubChem 2019 update: improved access to chemical data. *Nucleic Acids Res.* 47 D1102–D1109. 30371825 10.1093/nar/gky1033 PMC6324075

[B26] Li J. H. Liu S. Zhou H. Qu L. H. Yang J. H. (2014a). starBase v2.0*: decoding miRNA-ceRNA, miRNA-ncRNA and protein-RNA interaction networks from large-scale CLIP-Seq data*. *Nucleic Acids Res.* 42 D92–D97. 24297251 10.1093/nar/gkt1248 PMC3964941

[B27] Li J. Gao C. Wang Y. Ma W. Tu J. Wang J. (2014b). A bioinformatics method for predicting long noncoding RNAs associated with vascular disease. *Sci. China Life Sci.* 57 852–857. 10.1007/s11427-014-4692-4 25104459

[B28] Li L. Zhuang Y. Zhao X. Li X. (2018). Long Non-coding RNA in Neuronal Development and Neurological Disorders. *Front. Genet.* 9:744. 10.3389/fgene.2018.00744 30728830 PMC6351443

[B29] Loomes R. Hull L. Mandy W. P. L. (2017). What Is the Male-to-Female Ratio in Autism Spectrum Disorder? A Systematic Review and Meta-Analysis. *J. Am. Acad. Child. Adolesc. Psychiatry* 56 466–474. 10.1016/j.jaac.2017.03.013 28545751

[B30] Luo T. Liu P. Wang X. Y. Li L. Z. Zhao L. P. Huang J. (2018). Effect of the autism-associated lncRNA Shank2-AS on architecture and growth of neurons. *J. Cell. Biochem.* 120:30160788. 10.1002/jcb.27471 30160788

[B31] Ma X. Chen K. Lu Z. Piechowicz M. Liu Q. Wu J. (2019). Disruption of MET Receptor Tyrosine Kinase, an Autism Risk Factor, Impairs Developmental Synaptic Plasticity in the Hippocampus. *Dev. Neurobiol.* 79 36–50. 10.1002/dneu.22645 30304576 PMC6397659

[B32] Marchese F. P. Raimondi I. Huarte M. (2017). The multidimensional mechanisms of long noncoding RNA function. *Genome Biol.* 18:206. 10.1186/s13059-017-1348-2 PMC5663108 29084573

[B33] Martelle J. L. Nader M. A. (2008). A review of the discovery, pharmacological characterization, and behavioral effects of the dopamine D2-like receptor antagonist eticlopride. *CNS Neurosci. Ther.* 14 248–262. 10.1111/j.1755-5949.2008.00047.x 18801115 PMC2753830

[B34] Mercer T. R. Mattick J. S. (2013). Structure and function of long noncoding RNAs in epigenetic regulation. *Nat. Struct. Mol. Biol.* 20 300–307. 10.1038/nsmb.2480 23463315

[B35] Ornoy A. Weinstein-Fudim L. Ergaz Z. (2019). Prevention or Amelioration of Autism-Like Symptoms in Animal Models: Will it Bring Us Closer to Treating Human ASD? *Int. J. Mol. Sci.* 20:1074. 10.3390/ijms20051074 30832249 PMC6429371

[B36] Parikshak N. N. Swarup V. Belgard T. G. Irimia M. Ramaswami G. Gandal M. J. (2016). Genome-wide changes in lncRNA, splicing, and regional gene expression patterns in autism. *Nature* 540 423–427. 10.1038/nature20612 27919067 PMC7102905

[B37] Pereanu W. Larsen E. C. Das I. Estévez M. A. Sarkar A. A. Spring-Pearson S. (2018). AutDB: a platform to decode the genetic architecture of autism. *Nucleic Acids Res.* 46 D1049–D1054. 29186576 10.1093/nar/gkx1093 PMC5753210

[B38] Pinto D. Pagnamenta A. T. Klei L. Anney R. Merico D. Regan R. (2010). Functional impact of global rare copy number variation in autism spectrum disorders. *Nature* 466 368–372. 20531469 10.1038/nature09146 PMC3021798

[B39] Pramparo T. Lombardo M. V. Campbell K. Barnes C. C. Marinero S. Solso S. (2015). Cell cycle networks link gene expression dysregulation, mutation, and brain maldevelopment in autistic toddlers. *Mol. Syst. Biol.* 11:841. 10.15252/msb.20156108 26668231 PMC4704485

[B40] Purcell A. E. Jeon O. H. Zimmerman A. W. Blue M. E. Pevsner J. (2001). Postmortem brain abnormalities of the glutamate neurotransmitter system in autism. *Neurology* 57 1618–1628. 10.1212/wnl.57.9.1618 11706102

[B41] Quinlan A. R. Hall I. M. (2010). BEDTools: a flexible suite of utilities for comparing genomic features. *Bioinformatics* 26 841–842. 10.1093/bioinformatics/btq033 20110278 PMC2832824

[B42] Rossignol D. A. Frye R. E. (2012). A review of research trends in physiological abnormalities in autism spectrum disorders: immune dysregulation, inflammation, oxidative stress, mitochondrial dysfunction and environmental toxicant exposures. *Mol. Psychiatry* 17 389–401. 10.1038/mp.2011.165 22143005 PMC3317062

[B43] Saha A. Kim Y. Gewirtz A. D. H. Jo B. Gao C. Mcdowell I. C. (2017). Co-expression networks reveal the tissue-specific regulation of transcription and splicing. *Genome Res.* 27 1843–1858. 10.1101/gr.216721.116 29021288 PMC5668942

[B44] Scahill L. Mccracken J. T. King B. H. Rockhill C. Shah B. Politte L. (2015). Extended-Release Guanfacine for Hyperactivity in Children With Autism Spectrum Disorder. *Am. J. Psychiatry* 172 1197–1206. 26315981 10.1176/appi.ajp.2015.15010055

[B45] Tang J. Yu Y. Yang W. (2017). Long noncoding RNA and its contribution to autism spectrum disorders. *CNS Neurosci. Ther.* 23 645–656. 10.1111/cns.12710 28635106 PMC6492731

[B46] Tay Y. Rinn J. Pandolfi P. P. (2014). The multilayered complexity of ceRNA crosstalk and competition. *Nature* 505 344–352. 10.1038/nature12986 24429633 PMC4113481

[B47] Tong Z. Zhou Y. Wang J. (2020). Identification and functional analysis of long non-coding RNAs in autism spectrum disorders. *bioRxiv*10.3389/fgene.2020.00849 PMC7525012 33193567

[B48] Uchida S. Dimmeler S. (2015). Long noncoding RNAs in cardiovascular diseases. *Circ. Res.* 116 737–750. 10.1161/circresaha.116.302521 25677520

[B49] Wang Y. Zhao X. Ju W. Flory M. Zhong J. Jiang S. (2015). Genome-wide differential expression of synaptic long noncoding RNAs in autism spectrum disorder. *Transl. Psychiatry* 5:e660. 10.1038/tp.2015.144 26485544 PMC4930123

[B50] Willsey A. J. Sanders S. J. Li M. Dong S. Tebbenkamp A. T. Muhle R. A. (2013). Coexpression networks implicate human midfetal deep cortical projection neurons in the pathogenesis of autism. *Cell* 155 997–1007. 10.1016/j.cell.2013.10.020 24267886 PMC3995413

[B51] Wishart D. S. Feunang Y. D. Guo A. C. Lo E. J. Marcu A. Grant J. R. (2018). DrugBank 5.0*: a major update to the DrugBank database for* 2018. *Nucleic Acids Res.* 46 D1074–D1082. 29126136 10.1093/nar/gkx1037 PMC5753335

[B52] Yanai I. Benjamin H. Shmoish M. Chalifa-Caspi V. Shklar M. Ophir R. (2005). Genome-wide midrange transcription profiles reveal expression level relationships in human tissue specification. *Bioinformatics* 21 650–659. 10.1093/bioinformatics/bti042 15388519

[B53] Yuen C. R. K. Merico D. Bookman M. Howe L. J. Thiruvahindrapuram B. (2017). Whole genome sequencing resource identifies 18 new candidate genes for autism spectrum disorder. *Nat. Neurosci.* 20 602–611. 28263302 10.1038/nn.4524 PMC5501701

[B54] Zeng H. Qiu C. Cui Q. (2015). Drug-Path: a database for drug-induced pathways. *Database* 2015:bav061. 10.1093/database/bav061 26130661 PMC4485432

[B55] Zeng P. Chen J. Meng Y. Zhou Y. Yang J. Cui Q. (2018). Defining Essentiality Score of Protein-Coding Genes and Long Noncoding RNAs. *Front. Genet* 9:380. 10.3389/fgene.2018.00380 30356729 PMC6189311

[B56] Zerbino D. R. Achuthan P. Akanni W. Amode M. R. Barrell D. Bhai J. (2018). Ensembl 2018. *Nucleic Acids Res.* 46 D754–D761. 29155950 10.1093/nar/gkx1098 PMC5753206

[B57] Zhang B. Horvath S. (2005). A general framework for weighted gene co-expression network analysis. *Stat. Appl. Genet. Mol. Biol.* 4:17. 10.2202/1544-6115.1128 16646834

[B58] Zhang L. Liu L. Wen Y. Ma M. Cheng S. Yang J. (2019). Genome-wide association study and identification of chromosomal enhancer maps in multiple brain regions related to autism spectrum disorder. *Autism. Res.* 12 26–32. 10.1002/aur.2001 30157312

[B59] Ziats M. N. Rennert O. M. (2013). Aberrant expression of long noncoding RNAs in autistic brain. *J. Mol. Neurosci.* 49 589–593. 10.1007/s12031-012-9880-8 22949041 PMC3566384

