# Supplementary material for: Identification and Functional Analysis of Long Non-coding RNAs in Autism Spectrum Disorders
Source: Front Genet. 2020 Sep 16;11:849. doi: 10.3389/fgene.2020.00849 (PMC7525012; doi:10.3389/fgene.2020.00849)
Supplement: Supplementary file 2 [file Data_Sheet_2.docx]

Supplementary Material

## Supplementary Figures


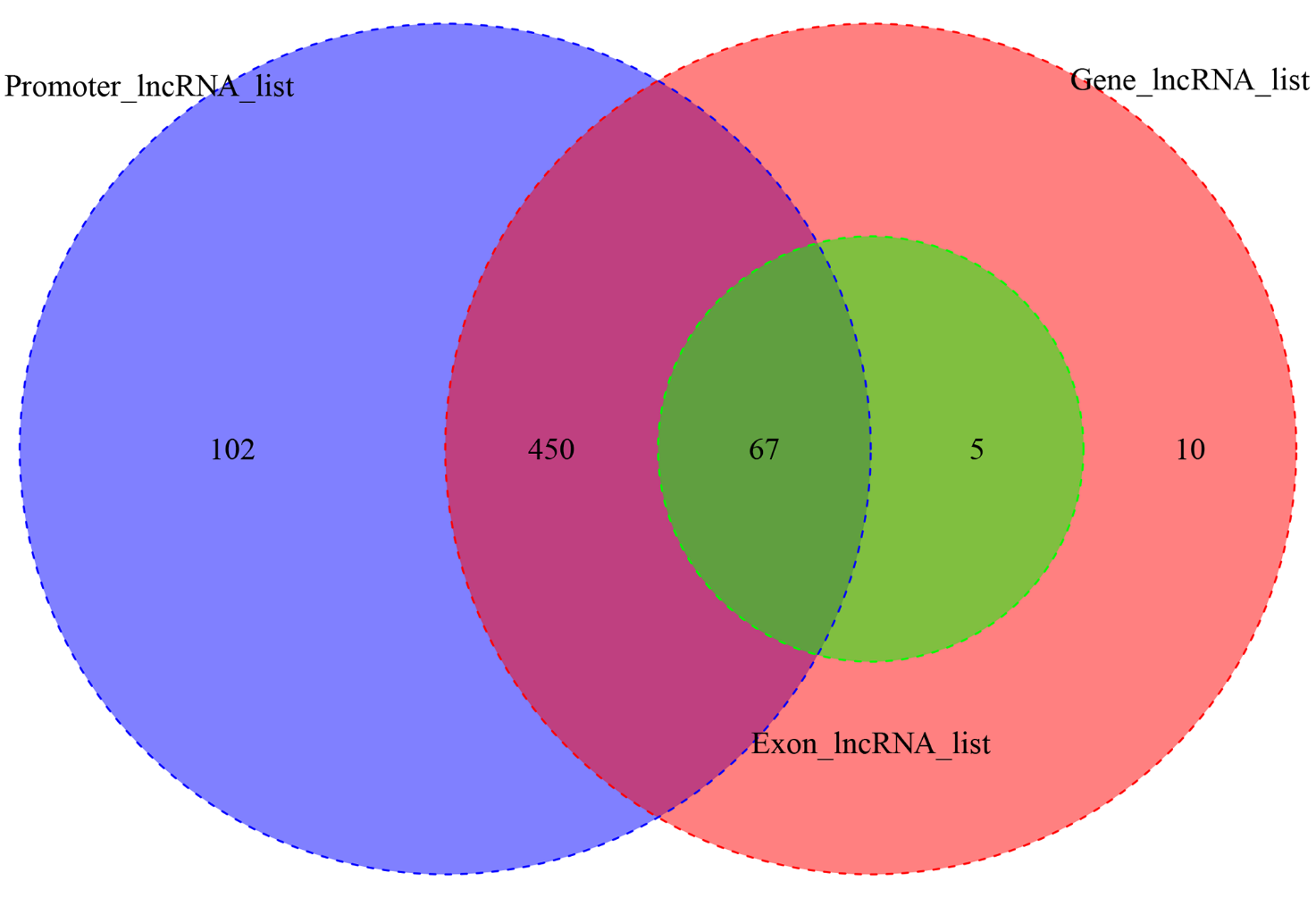


**Supplementary Figure 1.** The ASD-related lncRNA lists identified by using different genomic annotations of lncRNAs. Venn plot displays the intersection of the ASD-related lncRNA lists identified by using lncRNA genes, lncRNA exons and lncRNA genes with promoters as reference genomic annotations of lncRNAs.


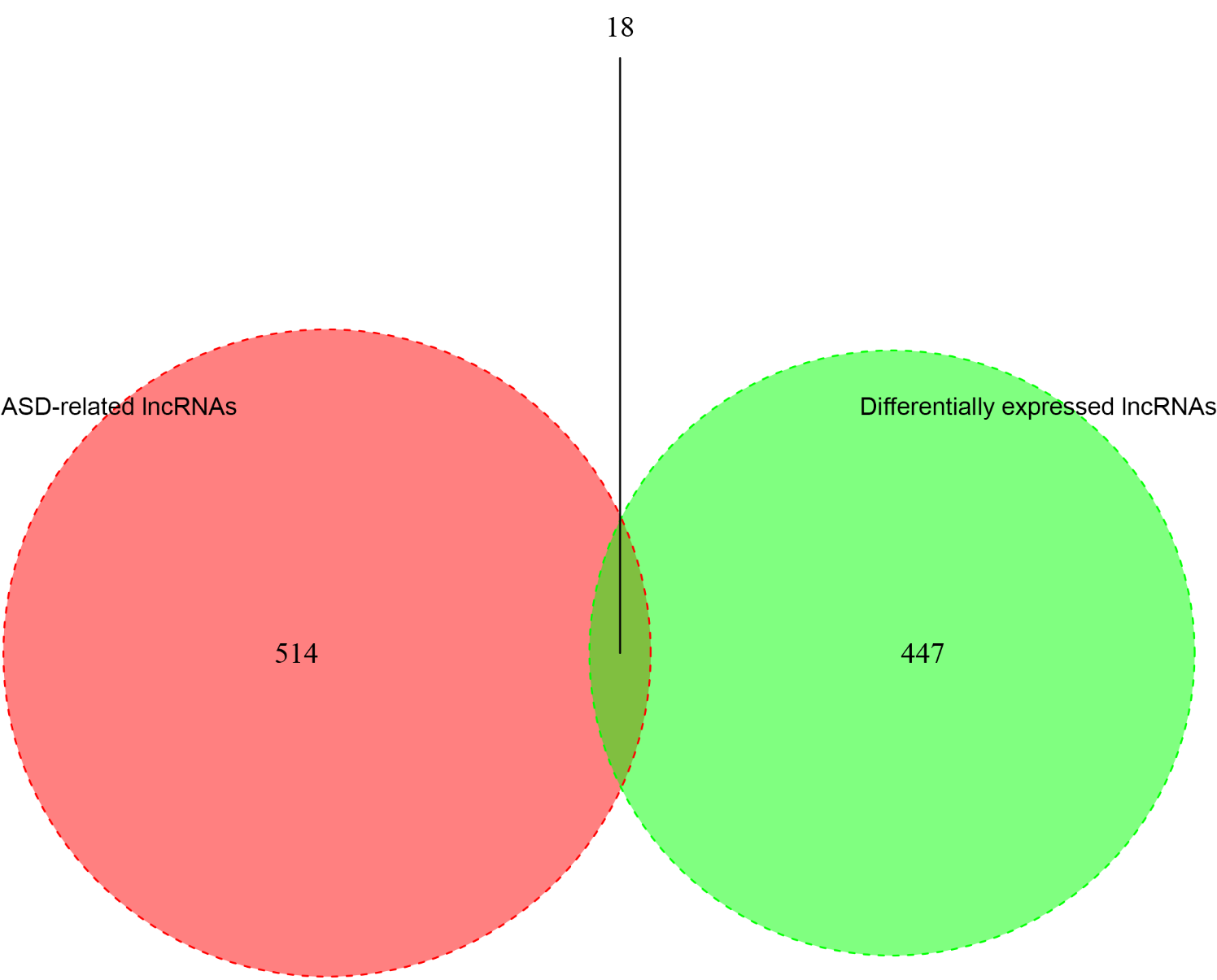


**Supplementary Figure 2.** The overlapping relationships between ASD-related lncRNAs and differential expressed lncRNAs in ASD. Red and green represent genomic variants-derived ASD-related lncRNAs and differentially expressed lncRNAs in the brain tissues in ASD, respectively.


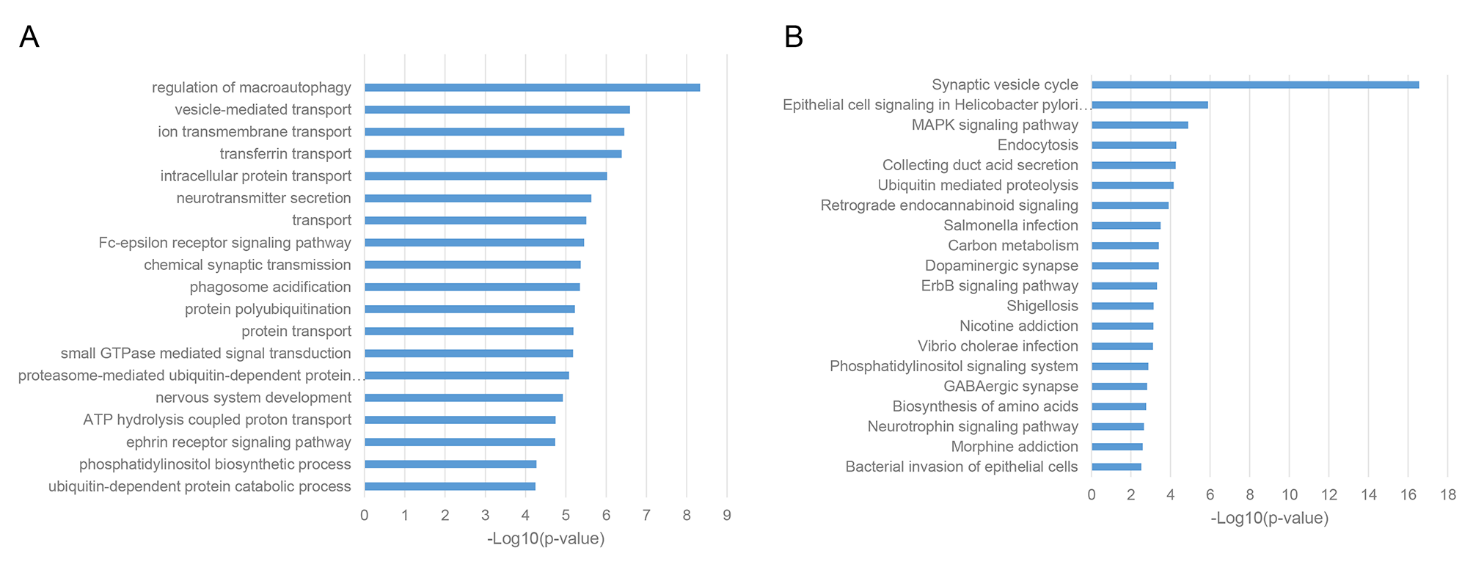


**Supplementary Figure 3.** Enriched functional terms of gene co-expression neighbors of the overlapping ASD-related lncRNAs with differentially expressed lncRNAs in the brain tissues in ASD. (A) Top 20 GO BP terms with p-value < 0.05. (B) Top 20 KEGG pathway terms with p-value < 0.05.


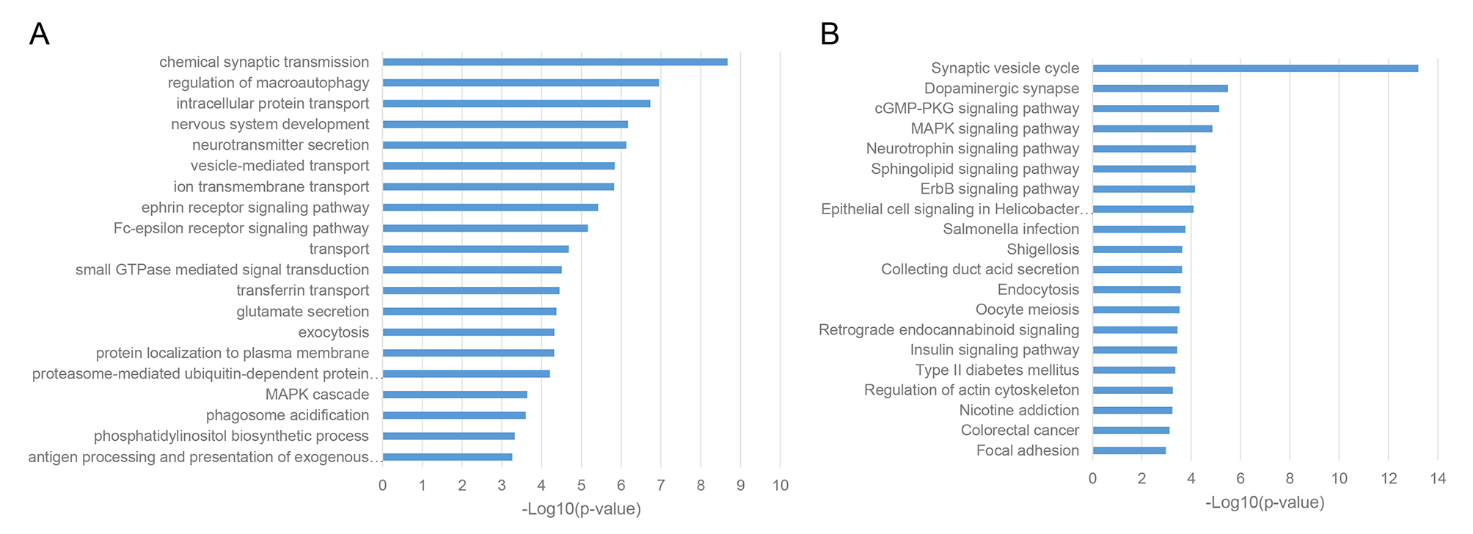


**Supplementary Figure 4.** Enriched functional terms of gene co-expression neighbors of the non-overlapping ASD-related lncRNAs with differentially expressed lncRNAs in the brain tissues in ASD. (A) Top 20 GO BP terms with p-value < 0.05. (B) Top 20 KEGG pathway terms with p-value < 0.05.
